# Identification of commercial *Ganoderma* (*Lingzhi*) species by ITS2 sequences

**DOI:** 10.1186/s13020-015-0056-7

**Published:** 2015-08-19

**Authors:** Baosheng Liao, Xiaochen Chen, Jianping Han, Yang Dan, Lili Wang, Wenjing Jiao, Jingyuan Song, Shilin Chen

**Affiliations:** Institute of Medicinal Plant Development, Chinese Academy of Medical Sciences and Peking Union Medical College, Beijing, 100193 People’s Republic of China; Institute of Chinese Materia Medica, China Academy of Chinese Medical Sciences, Beijing, 100700 People’s Republic of China

## Abstract

**Background:**

DNA barcoding can be used to authenticate *Ganoderma* species for safe use. This study aims to identify commercial products containing *Ganoderma* using DNA barcoding.

**Methods:**

We used 63 internal transcribed spacer (ITS) 2 sequences of *Ganoderma* species from 33 newly-sequenced wild samples, crude drugs, mycelia, spores, and authentic extracts and spore oils collected from various locations and markets, as well as 30 sequences from GenBank. Sequences were assembled and aligned using CodonCode Aligner V3.71. Intra- and inter-specific distances were estimated by MEGA 6.0, and phylogeny reconstruction was based on the K2P model. SNP(s) and RNA secondary structure of ITS2 were analyzed and compared among closely related *Ganoderma* species.

**Results:**

*G. lucidum* cultivated in China was different from those cultivated in Europe. “*Chizhi*” (*G. lucidum*) and “*Zizhi*” (*G. sinense*) were clustered into two clades that were separated from the other *Ganoderma* species. The fruiting bodies and commercial products of *G. lucidum* and *G. sinense* were successfully distinguished from those of other *Ganoderma* species by comparing the ITS2 sequences and RNA secondary structures.

**Conclusion:**

The DNA barcoding method is applicable to the authentication of commercial products containing *Ganoderma* species.

## Background

*Ganoderma* (*Lingzhi*) is widely used in health products for its anti-tumor, anti-aging, anti-bacterial, immune system-enhancing, and anti-hypertension activities [1–5]. *Lingzhi* and its derivative products have a world trade value of approximately four billion US dollars [6]. *Lingzhi* products are popular in the market because of their high demand and potential profits.

There are approximately 76 *Ganoderma* species in China [7], but only approximately 20 of the species are used for medical purposes [8]. Moreover, only *Ganoderma lucidum* (Leyss. ex Fr.) Karst., 1881 (*Chizhi*) and *G. sinense* Zhao, Xu et Zhang, 1979 (*Zizhi*) are officially described in the Chinese Pharmacopoeia [9], and they are the most common types of *Lingzhi* on the market. They are difficult to distinguish because of the intra-species diversity of morphological features [8].

*G. lucidum* was collected from the UK. Karsten (1881) established the genus *Ganoderma* based on *G*. *lucidum* [10], which was reported in China in 1934 and was first successfully artificially cultivated in 1969 [11]. Cao et al. [11] proposed a new species name, *G. lingzhi* in 2012 for the *Lingzhi* that is distributed in East Asia. However, Wang et al. [12] determined that the widely cultivated *G. lucidum* in China was, in fact, *G. sichuanense* based on morphological and molecular evidence. Although they provided descriptions for the Lingzhi species in China, they did not obtain sequences from type specimens of *G.sichuanense*. The genome sequence of *G. lucidum* in China was first published by our research group [13]. The taxonomy of *Lingzhi* in China is still under dispute.

The internal transcribed spacer (ITS) region was proposed as a global DNA barcode sequence for identification of fungi at the fourth International Barcode of Life Conference [14]. Chen et al. [15] proposed the nuclear ribosomal DNA second internal transcribed spacer (ITS2) locus as a novel universal DNA barcode to identify herbs based on 6600 samples that represented 4800 species. Han et al. [16] compared the ITS and ITS2 regions and found that ITS2 was more suitable for species identification because of its short length and high efficiency for PCR amplification of this region. Moreover, the sequences and secondary structures of ITS2 could be considered as molecular morphological characteristics for species identification [17]. Considering DNA degradation in Lingzhi products, especially Lingzhi extracts and spore oil, the shorter sequence of ITS2 would likely provide a higher amplification and identification efficiency.

This study aims to authenticate commercial products containing *Ganoderma* using the DNA barcoding method.

## Methods

### Sample collection and data acquisition

Sixty-three specimens belonging to 11 *Ganoderma* species were analyzed. Specimens included 33 samples of commercially cultivated fruiting bodies, strains, slices, spore powders, extracts and spore oils collected in this study, and 30 sequences obtained from GenBank (Table 1). Twenty-six samples of *G. lucidum*, five strains of *G. sinense*, and two samples of *G. resinaceum* were collected in this study. Voucher samples were deposited in the herbarium of the Institute of Medicinal Development at the Chinese Academy of Medicinal Science, Beijing, China. Other published *Ganoderma* ITS2 sequences were downloaded from GenBank and were also analyzed for their ability to identify species in this study. We screened 348 ITS sequences named *G. lucidum* (or *G. lingzhi*). Sequences that met the following criteria were selected: (1) the sequences had already been published; (2) the sequences had complete ITS2 regions; and (3) sequences with original samples that were not from East Asia and Europe or if the original location of the sample was unknown, would be abandoned. The original samples of European *G. lucidum* for ITS2 sequences that we selected were identified based on morphological features by Yun Cao during previous *Ganoderma* research [11] and were stored in the Mycological Herbarium, Institute of Microbiology, Chinese Academy of Sciences (HMAS).

**Table 1:** Species used in this study along with their species/strain numbers, geographic origins, and GenBank accession numbers

Species	Species/strain numbers	Geographic origin	Sample type	GenBank no.	References
*Ganoderma applanatum*	ATCC44053	Japan	–	JQ520161	[41]
*G. applanatum*	GA117	Jilin, China	–	DQ424996	[42]
*G. fornicatuma*	AS 5.539, Type 1	Taiwan, China	–	AY593859	[43]
*G. fornicatuma*	AS 5.539, Type 2	Taiwan, China	–	AY593860	[43]
*G. sinense* (*G. japonicum*)	AS 5.69, Type 1	Hainan, China	–	AY593864	[43]
*G. sinense* (*G. japonicum*)	AS 5.69, Type 2	Hainan, China	–	AY593865	[43]
*G. lucidum*	Dai12573	Liaoning, China	–	JQ781855	[11]
*G. lucidum*	SN04MT01	Heilongjiang, China	Fruiting body	KJ453526	This study
*G. lucidum*	SN04MT02	Heilongjiang, China	Fruiting body	KJ453527	This study
*G. lucidum*	SN04MT03	Shandong, China	Fruiting body	KJ453528	This study
*G. lucidum*	SN04MT04	Shandong, China	Fruiting body	KJ453529	This study
*G. lucidum*	SN04MT05	Shandong, China	Fruiting body	KJ453530	This study
*G. lucidum*	SN04MT06	Shandong, China	Fruiting body	KJ453531	This study
*G. lucidum*	SN04MT07	Shandong, China	Fruiting body	KJ453532	This study
*G. lucidum*	SN04MT08	Shandong, China	Fruiting body	KJ453533	This study
*G. lucidum*	SN04MT09	Shandong, China	Fruiting body	KJ453534	This study
*G. lucidum*	SN04MT10	Tianjin, China	Fruiting body	KJ453535	This study
*G. lucidum*	SN04MT11	Tianjin, China	Fruiting body	KJ453536	This study
*G. lucidum*	SN04MT12	Tianjin, China	Fruiting body	KJ453537	This study
*G. lucidum*	SN04MT13	Guangdong, China	Fruiting body	KJ453538	This study
*G. lucidum*	SN04MT14	Guangdong, China	Fruiting body	KJ453539	This study
*G. lucidum*	SN04MT15	Unknown, Shop	Medicinal slices	KJ453540	This study
*G. lucidum*	SN04MT16	Hebei, China	Fruiting body	KJ453541	This study
*G. lucidum*	SN04MT17	Hebei, China	Fruiting body	KJ453542	This study
*G. lucidum*	SN04MT18	Shandong, China	Fruiting body	KJ453543	This study
*G. lucidum*	SN04MT19	Shandong, China	Medicinal slices	KJ453544	This study
*G. lucidum*	SN04MT20	IMPLAD, China	Strain	KJ453545	This study
*G. lucidum*	SN04MT21	IMPLAD, China	Spores	KJ453546	This study
*G. lucidum*	SN04MT22	IMPLAD, China	Extract	KJ453547	This study
*G. lucidum*	SN04MT23	IMPLAD, China	Spore Oil	KJ453548	This study
*G. lucidum*	SN04MT24	Taiwan, China	Fruiting body	KJ453549	This study
*G. lucidum*	SN04MT25	Taiwan, China	Fruiting body	KJ453550	This study
*G. lucidum*	SN04MT26	Taiwan, China	Fruiting body	KJ453551	This study
*G. lucidum*	ASI-7004	Korea	–	JQ520167	[41]
*G. lucidum*	GlCN04	Italy	–	AM906058	[44]
*G. lucidum*	Dai2272	Sweden	–	JQ781851	[11]
*G. lucidum*	Dai11593	Finland	–	JQ781852	[11]
*G. lucidum*	CBS 270.81	France	–	Z37099	[45]
*G. lucidum*	HMAS 86597	U.K.	–	AY884176	[12]
*G. multipileum*	Dai9521	Hainan, China	–	JQ781874	[11]
*G. multipileum*	HMAS 242384	Sichuan, China	–	JF915409	[12]
*G. resinaceum*	DP2	Italy	–	AM906060	[44]
*G. resinaceum*	CBS 220.36	USA	–	JQ520201	[31]
*G. resinaceum*	SN06MT01	Shandong, China	Fruiting body	KJ453552	This study
*G. resinaceum*	SN06MT02	Shandong, China	Fruiting body	KJ453553	This study
*G. sichuanense*	HMAS42798 (holotype)	Sichuan, China	–	JQ781877	[11]
*G. sichuanense*	Cui7691	Guangdong, China	–	JQ781878	[11]
*G. sinense*	SN05MT01 (CGMCC5.0069, Type 1)	HMAS, China	Strain	KJ453554	This study
*G. sinense*	SN05MT02 (CGMCC5.0069, Type 2)	HMAS, China	Strain	KJ453555	This study
*G. sinense*	SN05MT03 (CGMCC5.0069, Type 3)	HMAS, China	Strain	KJ453556	This study
*G. sinense*	SN05MT04 (CGMCC5.0069, Type 4)	HMAS, China	Strain	KJ453557	This study
*G. sinense*	SN05MT05 (CGMCC5.0069, Type 5)	HMAS, China	Strain	KJ453558	This study
*G. sinense*	GS111	Shandong, China	–	DQ424995	[42]
*G. sinense*	GS92	Hubei, China	–	DQ424982	[42]
*G. tenue*	GTEN24, Type 1	Shanghai, China	–	DQ424977	[42]
*G. tenue*	GTEN24, Type 2	Shanghai, China	–	DQ424978	[42]
*G. tropicum*	Dai9724	Hainan, China	–	JQ781879	[11]
*G. tropicum*	Yuan3490	Yunnan, China	–	JQ781880	[11]
*G. tropicum*	HMAS 263143	Hainan, China	–	JF915410	[12]
*G. weberianum*	CBS 219.36	Philippines	–	JQ520219	[31]
*G. weberianum*	HMAS 97365	Hainan, China	–	JF915411	[12]
*Tomophagus colossus*	CGMCC 5.763	Philippines	–	JQ081068	[12]
*T. colossus*	ANH s.n.	Vietnam	–	JN184395	[46]

### DNA extraction, PCR amplification, cloning and sequencing

Specimens were divided into three groups. One group included fruiting bodies, slices, spore powders and extracts. Samples of approximately 30 mg were needed and were ground into powder using a Retsch MM400 (Retsch Co., Germany). Strains (50 mg) were homogenized in liquid nitrogen. Spore oil (300 µL) was first centrifuged at 10,625×*g* for 10 min using a Sigma 1-14K (Sigma Co., Germany), and the pellet was used for DNA extraction. Total genomic DNA was subsequently extracted using the Plant Genomic DNA kit (Tiangen Biotech Co., China) following the recommended protocol. One pair of primers, 156 (5′-AACCATCGAGTCTTTGAACGC-3′) and 157 (5′-CCTTGTAAGTTTCTTTTCCTCC-3′), were designed for PCR amplification of the ITS2 region of *Ganoderma*. PCR was performed in 25-µL reaction mixtures, containing 12.5 µL of 2 × PCR buffer (Aidlab Biotechnologies Co., China), 1 µL of each PCR primer (2.5 µM), and 2 µL of DNA extract, and the total volume was adjusted to 25 µL with sterile deionized water. PCR amplification was conducted according to the following procedure: 94 °C for 5 min, 40 cycles of 94 °C for 30 s, 50 °C for 30 s, and 72 °C for 1 min, and a final extension at 72 °C for 10 min. PCR products were analyzed by electrophoresis in a 1 % agarose gel. The PCR products were purified using the PCR Purification Kit (Tiangen Biotech Co., China) and sequenced bidirectionally using a ABI 3730XL sequencer (Applied Biosystems Co., USA) based on the Sanger sequencing method at the Genome Center, Chinese Academy of Agricultural Sciences.

### Phylogenetic analysis

The sequences were edited and assembled manually using CodonCode Aligner V3.71 (CodonCode Co., USA). The new sequences obtained in this study were deposited in GenBank. ITS sequences from GenBank were annotated using the Hidden Markov model (HMM) [18] to obtain the ITS2 sequences. All ITS2 sequences were included in the phylogenetic analysis by MEGA 6.0 [19]. All of the sequences were aligned using the MUSCLE method [20]. A neighbor-joining (NJ) [21] tree was constructed with the following parameters: the bootstrap method was conducted with 1000 replicates, the substitution model was Kimura-2-parameter (K2P) [22], and gaps were treated as missing data (complete deletion). Maximum parsimony (MP) [23] trees were constructed with the following parameters: the bootstrap method was conducted with 1000 replicates, the MP search method was subtree pruning and regrafting [24], the number of initial trees was ten (random addition), and gaps were treated as missing data (complete deletion). Sequence divergence was also calculated using the K2P model, and gaps were treated as missing data. *Tomophagus colossus* was selected as the outgroup. The secondary structure of ITS2 was predicted at the ITS2 database website (http://www.its2.bioapps.biozentrum.uni-wuerzburg.de/) [25].

## Results

### ITS2 sequence analysis and intra- and inter-species variations

**Fig. 1:**
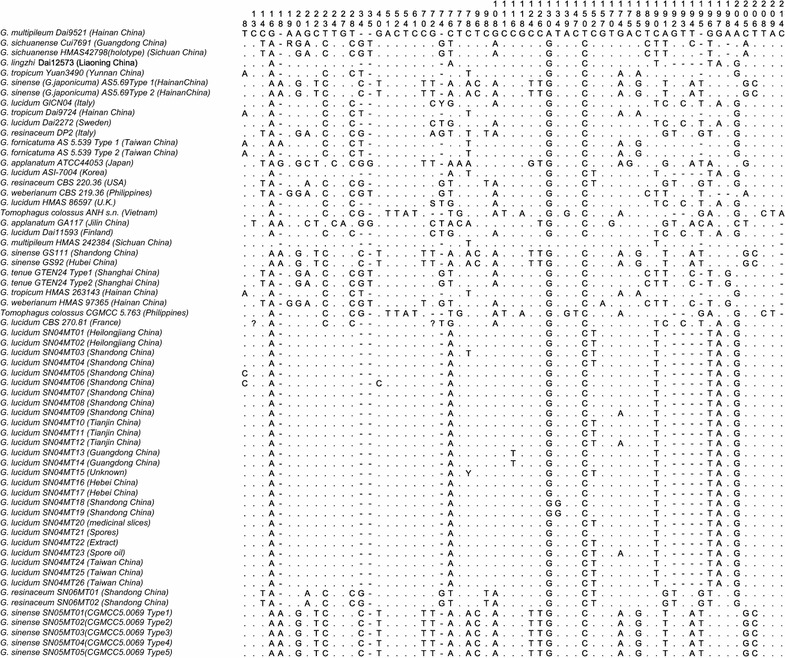
Multiple sequence alignment of 63 ITS2 sequences. The sequences were aligned using MUSCLE. Fifty-three variable sites in the 218-bp sequence alignment of the ITS2 were extracted and presented. The specimen names are shown in the *left side* of the alignment. Gaps are indicated with *dashes*, and identical sites are indicated with *dots*

The PCR product sizes for the ITS2 region ranged from 469 to 566 bp. The length of ITS2 was 218 bp after deletion of the 5.8S and 28S rDNAs and alignment using the MUSCLE method. The average G-C and A-T contents of the ITS2 region were 49.4 and 50.6 %, respectively. The aligned ITS2 rDNA sequences are shown in Fig. 1. The 26 newly collected samples of *G. lucidum* from China had seven intraspecific variable sites (Fig. 1), and 10 of these 26 samples had identical sequences. The ITS2 regions of *G. sinense* resulted in ambiguous sequences with direct sequencing of the PCR products; thus, a cloning method was used.

**Fig. 2:**
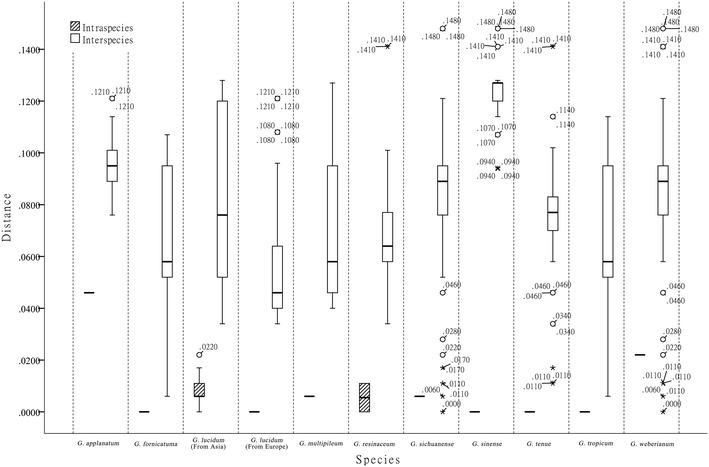
Intra- and inter-species distances. Intra- and inter-species distances of the ITS2 sequences of the *Ganoderma* genus based on the K2P model

Nucleotide analysis of the ITS2 region could provide more information about inter-and intra-species divergences. The average intraspecific genetic distances calculated by the Kimura-2-parameter model [22] were 0.007 for *G. lucidum* from East Asia. No variable sites were detected among the ITS2 regions of nine *G. sinense* samples collected from Shandong, Hubei and Hainan. The interspecific diversities ranged from 0.035 to 0.047 between *G. lucidum* from Europe and *G. lucidum* from East Asia, from 0.097 to 0.111 between *G. lucidum* from East Asia and *G. sinense*, and from 0.035 to 0.123 between *G. lucidum* from East Asia and the other species examined. In this study, the intra-species distances of the *Ganoderma* species were lower than the inter-species distances except for in *G. sichuanense* and *G. weberianum* (Fig. 2).

SNP-based molecular barcodes have been used for identification studies in closely related species [26]. There were seven stable SNPs existing between *G. lucidum* from East Asia and *G. lucidum* from Europe, including sites of deletion/insertion, and 25 stable SNPs between *G. lucidum* from East Asia and *G. sinense* including four sites of deletion/insertion (Fig. 1). At positions 23, 76, 108 and 196 bp, all *G. lucidum* samples from Europe contained C, G, A, and G, respectively. Meanwhile, all *G. lucidum* samples from East Asia contained T, A, G, and T, respectively.

### Phylogenetic analysis

Species of the genus *Ganoderma*, including *G. lucidum* (from Europe), *G. sinense*, *G. applanatum*, *G. fornicatuma*, *G. multipileum*, *G. resinaceum*, *G. sichuanense*, *G. weberianum*, *G. tenue*, and *G. tropicum*, which are closely related to *G. lucidum* (from East Asia), were used to study the relationships between Lingzhi species. Sixty-three ITS2 sequences were analyzed. A total of 218 characters were included for phylogenetic analysis, of which 61 were variable and 53 were parsimony informative characters. The consistency index was 0.6914, the retention index was 0.9324, and the composite index was 0.6614 for all sites and parsimony-informative sites (in parentheses).

**Fig. 3:**
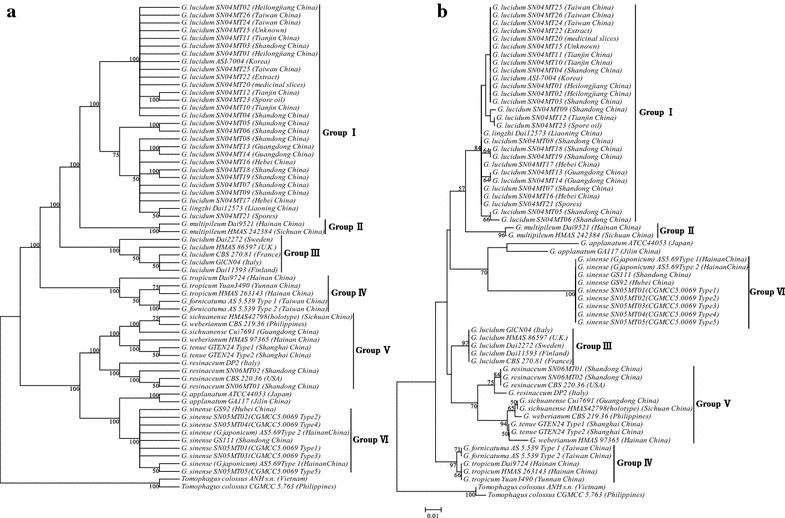
Phylogenetic tree based on the ITS2 region using the NJ method. Strict consensus phylogenetic trees constructed using MP (**a**) and NJ (**b**) methods based on the ITS2 sequences of 63 taxa of *Ganoderma*. Bootstrap values are shown above the branches. Based on the trees, the taxa can be divided into six clades

The topologies of the NJ and MP trees (Fig. 3) were similar. The high level of nucleotide substitution in the ITS2 rDNA resulted in six clades. Although most sequences of either *G. lucidum* or *G. sinense* had identical ITS2 sequences, *G. lucidum* from Europe did not fit into these clades. In the phylogenetic trees, Group 1 consists of collections from *G. lucidum* from East Asia; 28 sequences of *G. lucidum* from China and Korea and five sequences of *G. lucidum* from Europe were clustered into two distinct clades, which were separate from the other species with high bootstrap support values. The three unknown samples from Taiwan (SN04MT24, SN04MT25, and SN04MT26) clustered with *G. lucidum* from East Asia, and the Dai12573 strains of *G. lingzhi* were within the same group. These data strongly indicated that *G. lucidum* from East Asia was not the same species as *G. lucidum* from Europe, and that *G. lucidum* could be misnamed in Asia. Sequences of *G. sinense* and *G. japonicum* formed a high-support value clade (100 %). *G. sinense* Zhao, Xu et Zhang is a new species that Zhao et al. established in 1979 [27] to eliminate confusion with *G. japonicum* (Fr.) Lloyd. Our results confirmed that the two species should be synonymous because the ITS2 sequence of *G. sinense* was identical to that of *G. japonicum*.

Group 5 consisted of two subgroups (100 % bootstrapping): subgroup 5.1 included *G. sichuanense*, *G. weberianum* and *G. tenue*, and subgroup 5.2 consisted of four *G. resinaceum* samples. *G. sichuanense*, *G. weberianum*, and *G. tenue* were grouped into one well-supported clade (94.3 %), but the relationships among these species require further study. Two samples of *G. multipileum* from China clustered together and formed a clade with *G. lucidum* from Asia. *G. multipileum*, a species for which there was a holotype specimen from Taiwan, was suggested as the correct name for the tropical *Ganoderma* samples and showed a close relationship with *G. lucidum* from East Asia.

### Efficiency of species identification

BLAST1 was used to further evaluate the efficiency of ITS2. The barcode sequences obtained in this study were used to build corresponding reference sequence libraries as described previously [28]. The results showed that ITS2 successfully identified 100 % of the commercial *Lingzhi* products collected in this study.

**Fig. 4:**
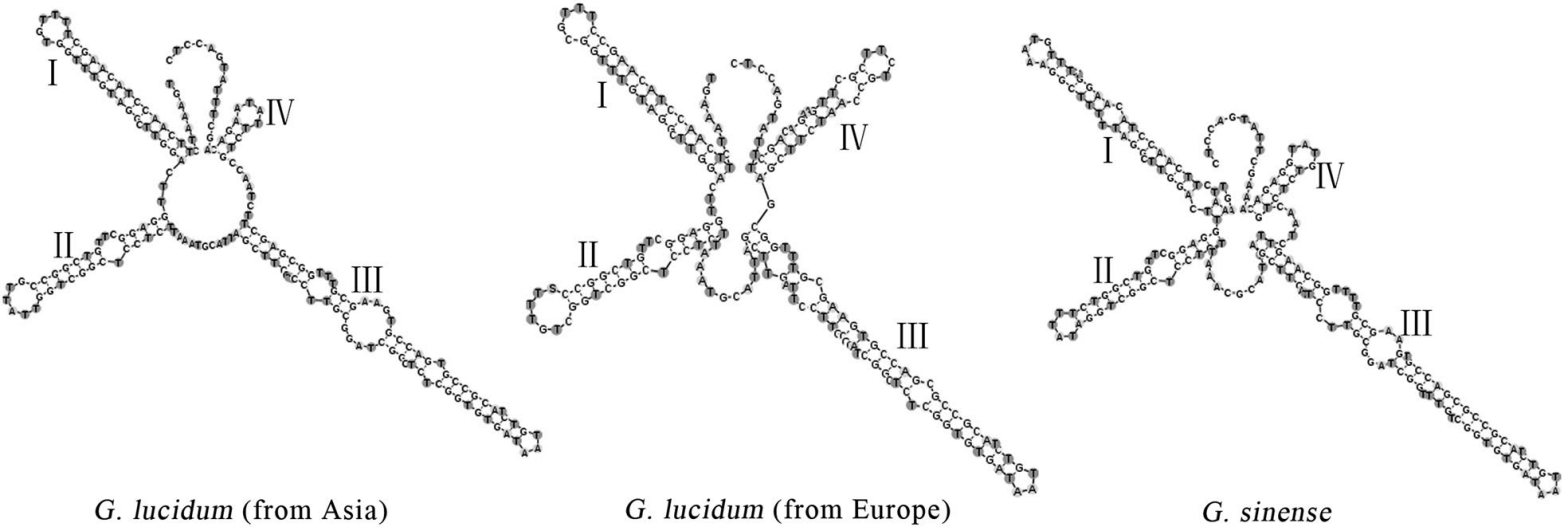
Secondary structures of ITS2 in three *Ganoderma* species. A four-fingered palm-like structure was observed. The four stem-loop domains are indicated with I–IV. The bulges are shown on each of the stem–loop domains

The ITS2 sequence-structure provided the most accurate phylogenetic analysis [29], and ITS2 sequence-structure information was correlated with the biological species concept [30]. Thus, the RNA secondary structures of ITS2 were analyzed to differentiate the species of *G. lucidum*. The three closely-related species have similar secondary structures of ITS2 sequences. Stem-loops I, II, and III were conserved, whereas stem-loop IV of the three species varied. The three species could be identified directly based on the RNA secondary structure of ITS2 (Fig. 4).

## Discussion

*G. lucidum* is one of the most economically important species of fungi; thus the stability of its taxonomy is highly important. Different researchers have different views on the scientific binomial of “*Chizhi*” [11, 12, 31–33]. In China, *Lingzhi* has long been misnamed *G. lucidum*. We identified commercial *Lingzhi* products based on the ITS2 rDNA marker, and our phylogenetic analysis clearly indicated that *Chizhi*, or *G. lucidum*, from East Asia, is not the same species as *G. lucidum* from Europe. The nucleotide divergence among *G. lucidum* from Europe and *G. lucidum* from East Asia, as well as the high bootstrapping support, indicated that they were different species. Analysis of RNA secondary structure further supported these results.

Identifying *Ganoderma* products (such as spore oil and extracts) according to morphological characteristics alone is difficult. The triterpenes of *G. lucidum* and *G. sinense* show significant differences in terms of types and content, and a distinction should be made between the medical uses of the two species [34–38]. The use of systematic methods for species identification and classification would be useful. The evolutionary context of the related species should be studied first to identify the biological species. An ideal barcode sequence should possess high inter-species divergence but low intra-species divergence to readily identify different species. ITS2 has a wider taxonomic coverage than was previously assumed because of the high sequence variability and conserved core secondary structure [16]. Moreover, ITS2 had comparable power for resolving closely related species, and especially for identifying herbs and specimens that have undergone DNA degradation. The nuclear ribosomal DNA second internal transcribed spacer ITS2 sequence is a double-edged tool for eukaryotic evolutionary comparisons [39], and has been proven useful for diagnostic purposes at the species level [40]. In the present study, we analyzed the ITS2 region of *Ganoderma* species to accurately identify commercial *Lingzhi* products. Our results showed that most *Ganoderma* products, including *Chizhi* (*G. lucidum*), *Zizhi* (*G. sinense*) and other *Ganoderma* species, could be successfully identified using ITS2 sequences. Our results also support the suggestion that *G. sinense* and *G. japonicum* should be considered synonymous because of their high sequence similarity.

In this study, regardless of the complicated taxonomy of *Ganoderma*, the sequence-based phylogeny supported the hypothesis that *G. lucidum* species originating in Europe and East Asia are not the same species.

## Conclusion

The DNA barcoding method is applicable to the authentication of commercial products containing *Ganoderma* species.
